# Doubly latent multilevel analysis of the relationship among collective teacher efficacy, school support, and organizational commitment

**DOI:** 10.3389/fpsyg.2022.1042798

**Published:** 2023-01-04

**Authors:** Zhonghua Zhang, John Chi-Kin Lee, Hongbiao Yin, Xin Yang

**Affiliations:** ^1^The Melbourne Graduate School of Education, The University of Melbourne, Melbourne, VIC, Australia; ^2^Department of Curriculum and Instruction, The Education University of Hong Kong, Tai Po, Hong Kong SAR, China; ^3^Department of Curriculum and Instruction, The Chinese University of Hong Kong, Shatin, Hong Kong SAR, China; ^4^Faculty of Education, Northwest Normal University, Lanzhou, China

**Keywords:** collective teacher efficacy, school support, organizational commitment, mediating effect, doubly latent multilevel analysis

## Abstract

**Introduction:**

Understanding the sources and the effects of collective teacher efficacy has been one of the central interests to many educational researchers and practitioners, because it is critical to understand how teachers can shape, and are shaped by, the educational processes in schools. Following the social cognitive perspective on the sources and consequences of efficacy beliefs, this study examined how school support influences collective teacher efficacy which in turn affects teachers’ organizational commitment.

**Method:**

The participants included 969 teachers sampled from 28 primary and secondary schools in Hong Kong. To appropriately address the nature of collective teacher efficacy and school support as school-level variables, the doubly latent multilevel structural equation modeling approach was used to analyze the data.

**Results:**

The results revealed the mediation mechanism played by collective teacher efficacy in explaining the effect of school support on teachers’ organizational commitment.

**Discussion:**

Schools are suggested to consider fostering a supportive school environment as a strategy to improve teachers’ collective efficacy beliefs if it is wished to enhance teachers’ commitment to schools.

## 1. Introduction

In recent years, policy makers, educators, and researchers have taken rapidly growing interests in the study of collective teacher efficacy (Donohoo, 2017; Cansoy et al., 2020; Qadach et al., 2020; Karacabey et al., 2022). In this era that emphasizes school improvement and accountability, it is of little surprise that efforts exerted by researchers and practitioners into searching for school characteristics that can lead to positive impact on student achievement have been significantly enhanced (Hoy et al., 2006). Of the school properties that have been identified to be helpful to explain differential performances between schools, collective teacher efficacy, which has been found to vary greatly among schools, has been suggested as a powerful construct that can advance a promising understanding of ways how schools can successfully foster student achievement (Goddard et al., 2000; Tschannen-Moran and Barr, 2004; Schechter and Tschannen-Moran, 2006; Donohoo, 2017).

Although the relationship between collective teacher efficacy and school conditions has been widely explored in the literature, many of the existing studies fail to appropriately deal with the nature of collective teacher efficacy and other school-level variables. Many studies also lack a clear theoretical foundation when formulating the relationship between collective teacher efficacy and other factors. To address these issues, the present study, following Bandura’s (1997, 2000), social cognitive theory, chose to adopt a doubly latent multilevel analysis to examine the relationship between collective teacher efficacy, school support, and teachers’ organizational commitment in Hong Kong schools.

## 2. Theoretical framework and hypotheses

### 2.1. The social cognitive perspective of efficacy beliefs

In social cognitive theory, Bandura (1997) differentiated two types of efficacy beliefs, namely, self-efficacy and collective efficacy: the former refers to the “beliefs in one’s capabilities” (p. 3), while the latter denotes “a group’s shared belief in its conjoint capabilities” (p. 477), to organize and execute the courses of action required to produce given levels attainments. Both types of efficacy beliefs have been suggested as the core mechanisms of human agency to deal with the challenges from the environments, and thus significantly influence individuals’ psychological and behavioral functioning (Bandura, 1997, 2000).

Bandura (1997) further defined the sources and consequences of efficacy beliefs. There are four major sources of efficacy beliefs in the social cognitive theory: (1) enactive mastery experiences, referring to individuals’ past attainments; (2) vicarious experiences, referring to the experiences derived from indirect, social learning of others’ performance; (3) social persuasion which is derived from the evaluative feedback from others; and (4) physiological and affective states that individuals partly rely on when judging their capabilities. Meanwhile, efficacy beliefs significantly influence the quality of human functioning in four aspects. Cognitively, efficacy beliefs influence individuals’ goal settings by directing attention and construal of environmental demands. Affectively, efficacy beliefs influence people’s capabilities to control or mobilize their emotions and deal with threatening environments. Motivationally, efficacy beliefs influence individuals’ the expectations of whether and what they can do to produce certain outcomes. Behaviorally, efficacy beliefs influence people’s decision and choice of activities and environments, because people avoid activities and situations they believe exceed their capabilities (Bandura, 1997).

Bandura’s (1997) social cognitive theory has dramatically influenced the research on teachers’ efficacy beliefs in the past decades. Educational researchers have extensively examined the roles of teachers’ perceived self-efficacy, or collective efficacy in impacting student learning, teaching effectiveness, and school improvement (e.g., Zee and Koomen, 2016; Donohoo, 2017). Various sources of teachers’ efficacy beliefs, such as teacher development programs, school leadership styles, and teacher and student conditions, have also been explored in the literature (e.g., Adams and Forsyth, 2006; Klassen et al., 2011; Morris et al., 2017). In the present study, we focus on teachers’ perceived collective efficacy beliefs, or collective teacher efficacy. The social cognitive perspective of the sources and consequences of efficacy beliefs provides us an appropriate framework to conceptualize the relationships between collective teacher efficacy, the perceived support in schools, and teachers’ organizational commitment.

### 2.2. Collective teacher efficacy

In line with the social cognitive theory, Goddard et al. (2000) defined collective teacher efficacy as a construct that measures teachers’ shared beliefs about the capability of their school as a faculty to have a positive impact on student achievement. It refers to teachers’ collective perceptions that their school as a whole can execute course of actions required to bring about desired ends, over and above the educational impact of their homes and communities (Tschannen-Moran and Barr, 2004; Schechter and Tschannen-Moran, 2006; Hoy, 2012). Collective teacher efficacy therefore essentially represents a characteristic of a school, reflecting the beliefs concerning the performance capacity of the school rather than the capabilities of individuals (Goddard, 2001; Goddard et al., 2004; Tschannen-Moran and Hoy, 2007; Takahashi, 2011; Moolenaar et al., 2012). As explained by Tschannen-Moran et al. (2014), collective teacher efficacy reflects “what teachers believe they as a group can accomplish, not what they as individuals can accomplish…” (p. 303). It implies that collective teacher efficacy, which represents an emergent attribute of a school, is conceptually distinct from the construct of self-efficacy, which reflects a trait of an individual teacher (Bandura, 1997; Goddard and Goddard, 2001; Yin et al., 2022). The nature of being defined as a school feature determines that collective teacher efficacy should be undertaken as a school-level factor to explain the differential effects that schools have on promoting teacher professionalism and nurturing student achievement (Goddard et al., 2000; Hoy, 2012; Moolenaar et al., 2012).

Knowledge of the sources and the effects of collective teacher efficacy is of interest to many educational researchers and practitioners because it is believed to be significantly critical for understanding how schools exert efforts to respond to educational policy (Evans, 2009; Donohoo, 2017). The four sources of efficacy beliefs suggested by Bandura (1997) are well applicable to the understanding of collective teacher efficacy (Goddard and Goddard, 2001). At a practical level, Adams and Forsyth (2006) asserted that collective teacher efficacy beliefs are subject to the effects of school contextual factors such as enabling school structure. These proximate sources, which include variables nested within the school contexts and exert day-in and day-out influences on teacher teaching and student learning, can also be salient in shaping collective teach efficacy (Adams and Forsyth, 2006; Tschannen-Moran et al., 2014).

Despite the impressive progress brought by the continuously prosperous studies, our understanding of how collective teacher efficacy is influenced by school contextual variables is still modest (Adams and Forsyth, 2006; Klassen et al., 2011). In addition, although studies have found that collective teacher efficacy is closely related to teachers’ commitment to profession (e.g., Tschannen-Moran et al., 2014) and students (e.g., Lee et al., 2011), there is a lack of empirical evidences on the positive effects of collective teacher efficacy on organizational commitment. In this investigation, we attempted to investigate how school support that the teachers experienced their daily works in the school influence teachers’ perceived collective efficacy beliefs which in turn impact their organizational commitment to schools.

### 2.3. School support and collective teacher efficacy

In the present study, school support refers to teachers’ perceptions of the support that they receive from their principals, colleagues, and the school structure. School support can been seen as a typical environmental factors which combines the four sources of efficacy beliefs suggested by Bandura (1997). Working in a more supportive school environment, teachers are more likely to observe the classes or participate in the workshops conducted by the experienced colleagues (various experiences); receive the professional guidance from their leaders and experienced colleagues (social persuasion); and practice what they learn from others in their own classrooms and receive the feedback from their peers (mastery experiences). Moreover, they could obtain the necessary materials and conditions to support their instructional practices, which helps relieve the negative feelings that they may experience in professional development (affective states). In this sense, teachers’ perceived school support may vary between schools.

When teachers are assessing their beliefs in their school’s capability of making a difference to their students, they will make use of past experiences and the current contextual circumstances of the school to conduct collective analyzes of the requirements of the anticipated tasks faced by the school and the assessment of the school’s capability (Adams and Forsyth, 2006; Tschannen-Moran et al., 2014). As part of the assessment of the requirements of teaching tasks, the availability of resources and support from the school are likely to play important roles in determining the hardness of tasks. Strong support from the school leader and colleagues can undermine a teacher’s evaluation of the obstacle of the teaching task and hence improve the confidence in solving the task with current teaching competence.

Indeed, Skaalvik and Skaalvik’s (2010) study found the strong positive correlation between collective teacher efficacy and supervisory support from the school leaders. Schools whose teachers reported high levels of collective efficacy beliefs tended to be those schools whose teachers also reported that their administrators, students, and parents were more supportive (Klassen et al., 2010). In this study, by using the doubly latent multilevel analysis to deal with the methodological issues that we raised above, we attempted to test the following hypothesis.

*H1*: School support has a positive impact on collective teacher efficacy.

### 2.4. Collective teacher efficacy and organizational commitment

The present study considers teachers’ organizational commitment as an indicator of the consequences of collective teacher efficacy on their professional functioning. Organizational commitment, which has been emerging as one of the central concepts in the field of organizational psychology over the past three decades (Dumay and Galand, 2012), can be defined as the identification of group members with the values and goals of the organization, the willingness to exert effort on behalf of the organization, and the desire to remain within or stay in the organization (Ross and Gray, 2006). In the school context, organizational commitment represents a psychological bond between teachers and a school, reflecting the degree to which a teacher feels a sense of loyalty to the school and the degree to which a teacher internalizes a school’s goals and shared values (Ware and Kitsantas, 2011; Dumay and Galand, 2012; Cansoy et al., 2020). It is important to understand the process through which teachers commit to their schools because organizational commitment has been identified as one of the vital predictors of teachers’ intention to quit from the school, job satisfaction, engagement in work behaviors, actual turnover in their teaching, and student achievement (Bentein et al., 2005; Guarino et al., 2006; Cansoy et al., 2020).

An individual’s commitment to an organization is formed based on the combination of his or her work experience and perceptions of the organizational and person characteristics (Dumay and Galand, 2012). Following the social cognitive theory, teachers’ organizational commitment mainly represents the affective and motivational effects that efficacy beliefs may have on teachers’ professional functioning toward school organization. Collective teacher efficacy, an emerging school property, is therefore expected to be a factor affecting teachers’ organizational commitment. Bandura (1997) asserted that collective teacher efficacy beliefs could engender a sense of shared missions of a school and strengthen teachers’ common commitments to achieve to accomplish the missions. In this sense, strong collective efficacy beliefs can lead to individual commitment to shared goals and commitment to collaboration with group members to reach these goals (Goddard et al., 2000; Takahashi, 2011). In this investigation, we used empirical data to examine whether the positive relationship between collective teacher efficacy and organizational commitment can be corroborated.

*H2*: Collective teacher efficacy has a positive effect on organizational commitment.

### 2.5. School support and organizational commitment

Given the role of teachers’ perceived organizational characteristics in forming their organizational commitment, it is not surprising that school support can exert a positive influence on organizational commitment (Dumay and Galand, 2012; Gündüz, 2014). Indeed, early studies have identified both sense of isolation from colleagues and supervisors and insufficient administrative support as sources of low levels of teacher commitment (Coladarci, 1992; Reyes and Pounder, 1993). Teachers who experience supportive feedback from school administrators and colleagues are not likely to feel increasingly isolated or ineffective (Gibbs and Miller, 2014). Their motivation and efficacy beliefs are less likely to be adversely affected and therefore they are more likely to exhibit commitment to teaching and schools (Ware and Kitsantas, 2007; Gündüz, 2014). Teachers’ organizational commitment can therefore be promoted by providing assistance and support directly to teachers to unite faculties toward the shared visions and goals of the school (Hulpia et al., 2011).

*H3*: School support has a positive effect on organizational commitment.

### 2.6. Collective teacher efficacy: A pathway on which how school support affects organizational commitment

As suggested by Bandura (1997), efficacy beliefs are the core mechanisms of human agency to deal with the challenges from the environments, and thus significantly influence individuals’ functioning. This implies that efficacy beliefs may mediate the effects of the environmental factors on individuals. Accordingly, we hypothesize that collective teacher efficacy acts as a mediator which partly explains the influences of school support on organizational commitment. Collective teacher efficacy has been interpreted as a motivational pathway on which how principal leadership influences a range of teacher outcomes including professional learning, commitment to school, and intent to leave (Dumay and Galand, 2012; Qadach et al., 2020; Karacabey et al., 2022). Similarly, in this empirical study, by testing the mediating role of collective teacher efficacy, we attempted to seek the support for taking collective teacher efficacy as one of the mechanisms through which school support influences teachers’ commitment to schools.

*H4*: The influence of school support on organizational commitment is positively mediated by collective teacher efficacy.

### 2.7. The cultural context of teaching and leadership in Hong Kong

The present study was conducted in primary and secondary schools in Hong Kong. In Hong Kong, teachers and leaders work in a cultural context which is saliently different from that in the Anglo-American countries. As a representative of Chinese societies, the Confucian values are respected and prevalently followed in Hong Kong. Therefore, the cultural context in Hong Kong is featured by a high power distance, a strong collectivist tradition, and a consensus on the importance of education and schooling for the personal and societal development (Walker and Dimmock, 1999; Hofstede et al., 2010). These cultural and contextual characteristics may influence principal leadership and the interactions between teachers and their colleagues in Hong Kong, which further influence teachers’ perceptions of school support, efficacy beliefs, and commitment to the school. For example, Walker and Dimmock (1999) summarized the five types dilemmas experienced by Hong Kong principals, and pointed out that all dilemmas appeared to be based in values conflicts linked to the Chinese cultural values such as hierarchy, harmony, seniority, and relationships.

In the context with distinctive cultural values, teachers in Hong Kong may have conflicting opinions about school leaders and other with higher professional status. On the one hand, the high power distance between the principal and teachers makes teachers feel perturbed in implementing instructional programs (Wong, 2017). On the other hand, although it is believed teachers should be provided adequate professional autonomy to teach, it has been revealed that some Hong Kong teachers preferred directives from their principals or curriculum leaders (Lee and Dimmock, 1999). The results of quantitative studies also demonstrate a complex picture with mixed findings on the relationship between of principal leadership and teacher outcomes. Fox example, Hallinger and Ko (2015) found a negative impact of principal leadership on practices in strategic direction and policy environment (an indicator of school environment), but a positive effect on teacher growth and development. Therefore, we cannot take for granted that school leadership or supportive school conditions necessarily have positive effects on teachers’ efficacy beliefs, as what have been revealed in the previous studies, most of which were conducted in Western cultural contexts.

In addition, Choi and Tang (2009) investigated the factors influencing Hong Kong teachers’ commitment during the period between 1997 and 2007 using a qualitative design. They summarized three types of influencing factors, namely, personal, workplace, and education system. The changes in any one of the three types of influencing factors may have implications for teacher commitment. Adopting a quantitative design, our study examined the effects of school-level influencing factors (i.e., school support and collective teacher efficacy) on teachers’ organizational commitment, with the purpose to provide suggestions for staff retention and teacher development in Hong Kong.

## 3. Doubly latent multilevel analysis

Another purpose of the study is to utilize a more robust statistical method than the traditional latent variable approaches such as confirmatory factor analysis (CFA) and structural equation modeling (SEM) to address the measurement issues relevant to level-specific latent constructs as well as the nested structure of the data. More specifically, given that all the three constructs are school-level constructs as well as the hierarchical structure of the data (i.e., teachers nested within schools), we utilized the doubly latent multilevel SEM approach (Marsh et al., 2009; Lüdtke et al., 2011; Muthén and Asparouhov, 2011) to test the aforementioned four research hypotheses that demonstrated the relationships among the three constructs. In the current study, all the three constructs, namely collective teacher efficacy, school support, and organizational commitment, are school-level *shared* latent constructs (Stapleton et al., 2016b) which were measured through a sample of individual teachers’ responses to a set of items. When a sample of individual teachers are selected from a school to evaluate a set of indictors tapping a school-level *shared* construct, the inherently nested nature of the data, the unit of analysis, as well as the need to control for both sampling and measurement errors arise the complications regarding the statistical methods (McNeish et al., 2017; Stapleton et al., 2016a, b). In such case, a synthesis of both the latent variable modeling method (e.g., CFA/SEM) well as the multilevel modeling method should be undertaken (Marsh et al., 2009). The recent development of doubly latent multilevel SEM approach (Lüdtke et al., 2011), which simultaneously integrates the latent variable approaches and the multilevel modeling method, offers the opportunity to deal with these methodological complexities. The latent variable approaches like CFA or SEM have traditionally been used to assess the support for a prior measurement or structural model with a control for measurement errors. Meanwhile, the multilevel modeling method has traditionally been applied to analyze the data with nested structure to correct test of statistical significance due to the inherent violations of assumption of sample independence and unconfound the effects of variables at different levels of analysis (Marsh et al., 2009, 2012). This feature of entailing integration of the latent variable approach and the multilevel modeling method enables the doubly latent multilevel SEM approach to provide the most comprehensive correction in terms of error variance when it comes to estimating group effects (Lüdtke et al., 2011). It offers new potential for the evaluation of school-level constructs like the three constructs examined in the study and has broad applicability to school organizational studies seeking to juxtapose the effects of individuals (e.g., teachers/students) and schools (Marsh et al., 2012). The technical presentations of how specific controls for measurement errors and sampling errors are implemented with the doubly latent multilevel SEM analysis can be found elsewhere (e.g., Marsh et al., 2009; Lüdtke et al., 2011; Muthén and Asparouhov, 2011; Stapleton et al., 2016a,b).

## 4. Materials and methods

### 4.1. Sample

Responses were collected from 969 teachers nested within 28 Hong Kong primary and secondary schools. These schools were involved in a large university and school partnership project led by the authors of this paper. The number of sampled teachers ranged from 7 to 59 in this sample, with an average of about 35 teachers per school.

### 4.2. Measures

Three measures were used in the present study to collect data. The wording of these scale items had been carefully adapted to make them suitable for the context of Hong Kong schools. Two of the measures (i.e., collective teacher efficacy scale and school support scale) had been applied to Hong Kong in previous research (Lee et al., 2011; Zhang and Yin, 2017).

*Collective teacher efficacy.* The collective teacher efficacy scale developed by Tschannen-Moran and Barr (2004) was used in this study. Following Bandura’s (1997) suggestion, this scale assesses teachers’ beliefs about the capacity of their school as a whole in teaching students and improving students’ learning achievements (Schechter and Tschannen-Moran, 2006). Twelve items were designed to measure two dimensions: *instructional strategies* (6 items) and *student discipline* (6 items). All the items were rated by the teachers on a 5-point scale (1 = nothing, 2 = very little, 3 = some degree, 4 = quite a bit, and 5 = a great deal). This instrument has been adapted in the Chinese context to assess Hong Kong teachers’ collective efficacy beliefs (Lee et al., 2011).

*School support.* Six items that were selected from the McREL Professional Learning Community Checklist (Lauer et al., 2005) were used to measure teachers’ perceived support from their schools. The teachers rated all of the items on a 4-point Likert scale ranging from 1 to 4 (1 = strongly disagree, 2 = disagree, 3 = agree, and 4 = strongly agree). This instrument has been adapted and applied to Hong Kong contexts in previous study (Zhang and Yin, 2017).

*Teacher commitment to school organization.* Teachers’ organizational commitment was measured by the three items used by Park (2005). The teachers evaluated each of the three items on a 6-point response scale to indicate their agreement (ranging from 1 = strongly disagree to 6 = strongly agree).

### 4.3. Analytical strategies

As argued above, to address the level-specific constructs and the nested structure of the data, the doubly latent multilevel SEM approach (Marsh et al., 2009; Lüdtke et al., 2011; Muthén and Asparouhov, 2011) was used to examine how school support, other than its direct effect, indirectly affects organizational commitment through collective teacher efficacy. Before testing the hypothesized structural model, the psychometric properties of the instruments measuring the three constructs were firstly examined. To utilize the doubly latent multilevel SEM technique to test the construct validity of the instruments, following the recommended guidelines for reporting multilevel SEM results (Kim et al., 2016), we conducted the analyzes in the following four steps.

The first step involved the preliminary analysis of the data. In the second step, two intra-class coefficients [ICC(1) and ICC(2)] were calculated for each item to justify the requirement of running multilevel analyzes. ICC(1) was used to detect whether there were significant and substantial variations located at the school level for each item (Raudenbush and Bryk, 2002). Large values of ICC(1) indicate substantial variations occurring at the school level, suggesting that multilevel analysis might be required to simultaneously incorporate both the teacher-level and school-level varieties. In this study, a value of ICC(1) greater than 0.05 warrants a multilevel analysis (Muthén and Satorra, 1995). Given that the responses of individual teachers within the same school were used to derive measures for school-level *shared* constructs (i.e., collective teacher efficacy, school support, and organizational commitment), ICC(2) was also estimated for each item. The values of ICC(2) provide the appropriateness of aggregating individual variables within groups to form a group-level shared variable. ICC(2) can be used as a measure of reliability of group-level component in multilevel models. ICC(2) values greater than 0.70 represent acceptable levels of reliability of a measured group-level shared construct (Stapleton et al., 2016b). In the third step, upon the indications of the values of ICC(1) and ICC(2) for the items, a series of doubly latent multilevel CFA analyzes were conducted to test the construct validity of the instrument. Given that all the three constructs are *cluster-level shared* constructs, they should be taken as pure school-level constructs in the analysis (Marsh et al., 2009; Morin et al., 2014; Stapleton et al., 2016b). Therefore, all of the multilevel CFA models tested in this step incorporated a saturated model at the teacher level and a hypothesized measurement model at the school level (*shared cluster construct MCFA model*; Stapleton et al., 2016b). In all the doubly latent multilevel CFA analyzes, the factor loading from each latent factor to the first indicator was fixed to 1 for model identification. In the last step, the composite reliability coefficients, which take into account the heterogeneous item-construct relations and the item-specific measurement errors, were calculated to estimate the amount of errors presented in the estimation of the latent constructs derived from the approved school-level measurement models (Geldhof et al., 2014).

The computer program Mplus Version 6.1 (Muthén and Muthén, 2008/2011) was used to run the analyzes. The maximum likelihood estimation with robust standard errors (MLR) method, which corrects the standard errors of the parameter estimates and the test of fit, was used to estimate the models (Rhemtulla et al., 2012). Missing data were handled using the full-information maximum likelihood method (Muthén and Muthén, 2008/2011).

The goodness-of-fit indices for model evaluation include the chi-square statistic χ^2^ , the comparative fit index (CFI), the Tucker-Lewis index (TLI), the root mean square error of approximation (RMSEA), and the standardized root mean residual (SRMR). In this study, model fit was judged as acceptable based on the criteria that the values of the CFI and the TLI were above 0.95 and the values of the RMSEA and the SRMR (i.e., SRMR_within_ for the teacher-level model and SRMR_between_ for the school-level model) were below 0.08 (Hu and Bentler, 1999; Marsh et al., 2004). To compare non-nested models, the Akaike information criterion (AIC) and the Bayesian information criterion (BIC) were used, with smaller AIC and BIC values being indicative of better fitting models.

## 5. Results

The means and standard deviations of the teachers’ responses to each of all the items are summarized in Table 1. The results indicated the positive attitudes of the teachers toward these indicators measuring the three constructs.

**Table 1:** Descriptive statistics, ICC(1), ICC(2), and standardized school-level factor loadings and the associated standard errors for items measuring collective teacher efficacy, school support, and organizational commitment.

Item	Mean	SD	ICC(1)	ICC(2)	Factor loading
Collective teacher efficacy (Scale score composite reliability ω = 0.982)					
1. How much can teachers in your school do to produce meaningful student learning?	3.636	0.593	0.111	0.812	0.975 (0.029)
2. How much can teachers in your school do to help students master complex content?	3.457	0.622	0.118	0.821	0.999 (0.025)
3. To what extent can school personnel in your school establish rules and procedures that facilitate learning?	3.522	0.638	0.090	0.775	0.997 (0.040)
4. How well can adults in your school get students to follow school rules?	3.603	0.717	0.192	0.892	0.845 (0.078)
5. How much can teachers in your school do to help students think critically?	3.289	0.686	0.126	0.833	0.998 (0.020)
6. How much can teachers in your school do to promote deep understanding of academic concepts?	3.450	0.685	0.105	0.803	0.963 (0.026)
7. How much can school personnel in your school do to control disruptive behavior?	3.570	0.737	0.132	0.841	0.915 (0.052)
8. To what extent can teachers in your school make expectations clear about appropriate student behavior?	3.676	0.615	0.117	0.819	0.999 (0.027)
9. How much can your school do to foster student creativity?	3.359	0.717	0.113	0.816	0.763 (0.155)
10. How much can your school do to get students to believe they can do well in schoolwork?	3.308	0.717	0.153	0.862	0.967 (0.029)
11. How well can teachers in your school respond to defiant students?	3.536	0.775	0.182	0.885	0.727 (0.135)
12. How much can your school do to help students feel safe while they are at school?	3.884	0.674	0.100	0.795	0.958 (0.032)
School support (Scale score composite reliability ω = 0.959)					
1. The principal consults staff before making decisions affecting them.	2.820	0.642	0.138	0.847	0.946 (0.053)
2. There is a formal support system at this school for beginning teachers.	2.794	0.615	0.168	0.874	0.853 (0.091)
3. Administrators facilitate teacher working together.	2.906	0.521	0.114	0.818	0.880 (0.075)
4. The principal ensures that teachers have the necessary materials to support high quality instruction.	2.882	0.500	0.119	0.825	0.868 (0.098)
5. Teachers are aware of what the principal believes regarding teaching and learning.	2.936	0.527	0.116	0.819	0.884 (0.056)
6. Administrators know the problems faced by staff.	2.704	0.634	0.077	0.743	0.933 (0.067)
Organizational commitment (Scale score composite reliability ω = 0.963)					
1. There is a great deal of cooperative effort among staff members.	4.720	0.788	0.102	0.798	0.904 (0.058)
2. There is broad agreement among the entire school faculty about the central mission of the school.	4.184	0.894	0.094	0.781	0.976 (0.043)
3. This school seems like a big family; everyone is so close and cordial.	4.187	1.028	0.121	0.826	0.954 (0.041)

The estimated ICC(1) values for all the items, which are shown in Table 1, range from 0.077 to 0.190, indicating the significant and nontrivial variances explained by the school level and warranting the multilevel analysis. The values of ICC(2), which can be found in Table 1, are all greater than 0.70. This provides support for the adequacy of aggregating the individual teachers’ responses to derive the measures for the school-level constructs for the three constructs at the school level.

**Figure 1:**
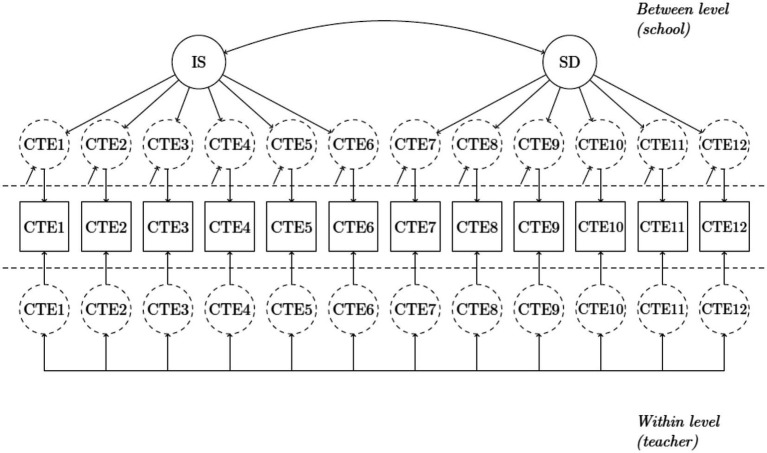
Two-factor measurement model (two school-level shared constructs). IS = instructional strategy; SD = student discipline; CTE = collective teacher efficacy.

The first measurement model (M1) that we tested for the construct of collective teacher efficacy was a doubly latent multilevel CFA model which incorporated a saturated model at the teacher level and a two-factor measurement model at the school level. This model is graphically depicted in Figure 1. The two factors in the school-level measurement model represented the two distinct but correlated school-level shared constructs: *instructional strategies* and *student discipline*. The results suggested that the residual variances of items 2 and 8 should be fixed to zero at the school level. In addition, the modification indices provided by the computer program suggested that allowing the residuals of item 4 with those of item 7 and item 11 as well as the residuals item 7 and item 11 to be correlated, respectively, could significantly improve the fit of the model. M1 was revised following these suggestions and was further tested. The goodness of fit indices suggested an adequate fit of the revised M1 (χ^2^ = 293.602, df = 50, CFI = 0.963; TLI = 0.903, and RMSEA = 0.071; SRMR_within_ = 0.002; SRMR_between_ = 0.090). The standardized estimates of the factor loadings of the school-level indicators are all greater than 0.762, suggesting strong relationships among the school-level indicators and their respective latent factors. The two school-level latent factors are extremely highly correlated (r=.978).

**Figure 2:**
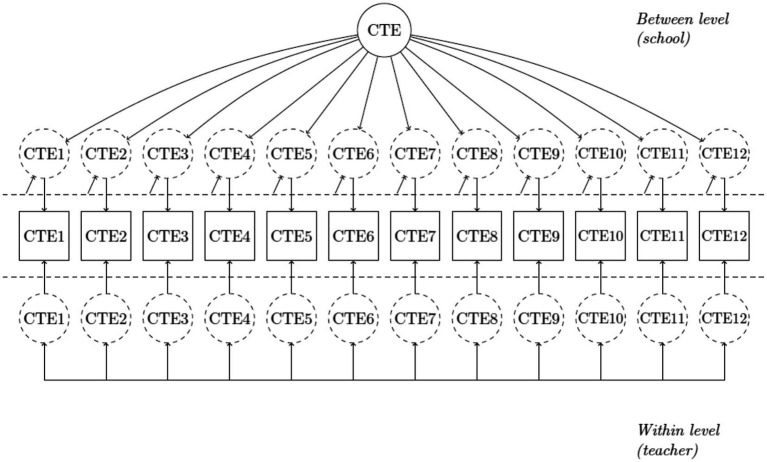
Single-factor measurement model (Single school-level shared construct). CTE = collective teacher efficacy.

Considering the extremely strong correlation between the two school-level latent factors, we further tested whether a single-factor measurement model (M2) could adequately represent the associations among the school-level indicators. As shown in Figure 2, M2 incorporated a saturated model at the teacher level and a single-factor measurement model at the school level. The modification indices also suggested to freely estimate the correlations between the residuals of item 4 and item 7, between the residuals of item 4 with item 11, and between the residuals of item 7 and item 11, respectively. A revised M2 based on the suggestions fitted the data reasonably (χ^2^ = 274.055, df = 51, and CFI = 0.966; TLI = 0.913, RMSEA = 0.067; SRMR_within_ = 0.002; SRMR_between_ = 0.090). The standardized coefficients of the factor loadings are presented in Table 1, supporting the construct validity of the scale.

The goodness of fit indices also provide support for acceptable model fit for school support (χ^2^ = 16.621, df = 9, and CFI = 0.994; TLI = 0.980, RMSEA = 0.030; SRMR_within_ = 0.001; SRMR_between_ = 0.044) and *Organizational commitment* (saturated model). The standardized estimates of the factor loadings are shown in Table 1, providing additional support for the adequacy of the construct validity for the two scales. It is noted that, given that all the items measuring school support were evaluated by the teachers on a four-point Likert scale, the construct validity of the instrument was also examined by conducting the doubly latent multilevel CFA analysis with the weighted least square mean and variance adjusted (WLSMV; Muthén and Muthén, 2008/2011) estimator by treating the data as ordinal data. In general, the results are consistent with those derived from the analysis using MLR estimator. The goodness of fit indices suggested the adequate fit of the measurement model (χ^2^ = 6.751, df = 9, and CFI = 1.00; TLI = 1.00, RMSEA = 0.00; SRMRwithin = 0.001; SRMRbetween = 0.054). The standardized factor loadings range from 0.840 to 0.912, which are very close to those obtained from the analysis using the MLR estimator.

The composite reliability coefficients of the scale scores for the three latent constructs are 0.982 (collective teacher efficacy), 0.959 (school support), and 0.963 (organizational commitment), respectively, indicating the sufficiently high levels of agreement between the indicators that were used to measure their, respectively, latent factors at the school level.

**Figure 3:**
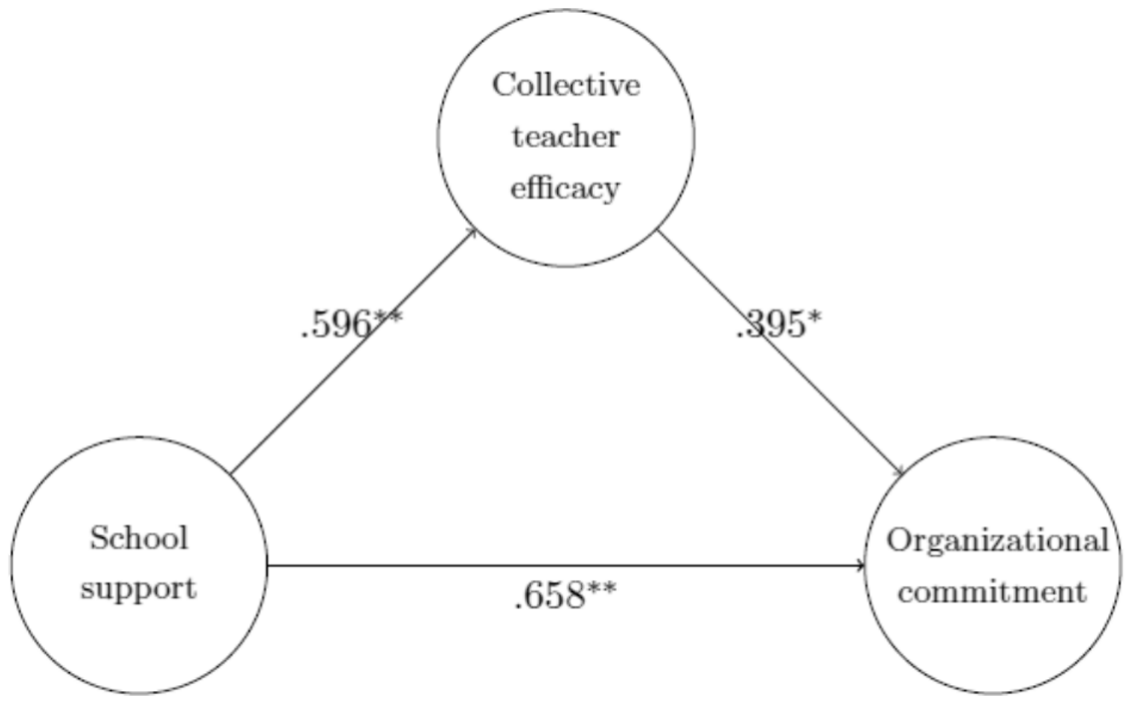
Doubly latent multilevel SEM analysis of the relationship among school support, collective teacher efficacy, and organizational commitment (school-level SEM model). ^*^p < 0.05; ^**^p < 0.01.

The standardized coefficients of the structural parameters that were estimated from the doubly latent multilevel SEM analysis are presented in Figure 3. As indicated by the results, school support was found to have a positive influence on collective teacher efficacy (β=.596,p<.01, and Hypothesis 1) which presented a positive direct effect on organizational commitment (β=.395,p<.05, and Hypothesis 2). In addition, school support also appeared to have a positive direct effect on organizational commitment (β=.658,p<.01, and Hypothesis 3). Moreover, the indirect effect of school support on organizational commitment through collective teacher efficacy was statistically significant and positive (β=.235,95%CI[.059,.411],p<.01, and Hypothesis 4), corroborating the mediating role played by collective teacher efficacy in the relationship between school support and organizational commitment.

## 6. Discussion

A better understanding of the sources and consequences of collective teacher efficacy is believed to be of great importance because it has been found to be highly correlated with teachers’ professional functioning. In this investigation, we set out to utilize the doubly latent multilevel method to explore the theoretical underpinnings for the interplay among the constructs of school support, collective teacher efficacy, and organizational commitment. Two points make the present study different from others. First, the study was clearly based on the social cognitive theory to formulate the relationship between the constructs of interest. Second, the adoption of doubly latent multilevel method help helps obtain some robust results on the relationship between them. The findings of the substantive model have important practical implications.

### 6.1. Effect of school support on collective teacher efficacy

The results of this study indicated the significant and positive impact of school support on collective teacher efficacy, highlighting the salient role of school support in shaping teachers’ sense of collective efficacy beliefs. This is not a surprising finding because, as Goddard (2001) stated, collective teacher efficacy is an emergent school characteristic that is formed based on the fact that teachers weigh their collective teaching competence, difficulties inherent in teaching facing the school, and the support available in the school setting. Teachers will usually weigh these information to eventually arrive at their beliefs about the possibility of achieving student learning (Goddard et al., 2000; Adams and Forsyth, 2006; Takahashi, 2011). Sufficient staff support enlisted from school will be likely to increase teachers’ confidence of overcoming barriers or limitations as well as the difficulties of tasks to pursue effective actions which can to lead to a positive outcome to student. Therefore, teachers who perceive the good availability of the necessary resources that they need to enact effective instruction in a socially supportive teaching environment tend to have higher confidence in the capacity of their colleagues with whom they work (Cybulski et al., 2005; Lim and Eo, 2014). However, teachers will feel discouraged if they realize that insufficient support and little professional development can be offered by their school when they are uncertain about their ability to make a difference to a given task (Cybulski et al., 2005). This finding is in line with the conclusions converged from previous studies. For example, Ross et al. (2004) identified the contribution of school cohesion and support to collective teacher efficacy in the domain in which the school had control over its direction. In their study of enabling schools, Adams and Forsyth (2006) found that promoting positive and healthy social interaction among teachers to generate positive collective teacher efficacy was dependent on a supportive and enabling school structure. In short, this finding suggests that by providing teachers with social support and the means necessary to effectively reduce job demands, collective efficacy can be enhanced, providing additional evidence that some school contextual characteristics can be proximate sources of boosting teachers’ collective efficacy beliefs.

### 6.2. Effect of collective teacher efficacy on organizational commitment

Our results demonstrated collective teacher efficacy a significant and positive predictor of teachers’ commitment to schools. This finding highlights collective teacher efficacy as an important school feature that is systematically related to organizational commitment, offering additional supportive evidence that the beliefs that the teachers hold can powerfully influence their commitment. This result implies that the teachers who are strongly confident in their school’s capability to successfully educate students are more committed to the success of the school and are willing to work beyond their assigned duties and responsibilities to ensure greater school success (Angelle and Teague, 2014). The fundamental assumption of social cognitive theory can provide an explanation for the positive influence of collective teacher efficacy on organizational commitment. Goddard et al. (2000) advocated that the effect of collective teacher efficacy on a school needs to be understood through how collective teacher efficacy shapes and changes the behavioral and normative environment of a school. The power of collective teacher efficacy lies in its ability of creating, shaping, and altering the norms and cultures of a school (Goddard, 2001). These social norms constituted by the shared beliefs among the teachers within the school can have social persuasion on teacher behaviors and exert influences on the actions of the school (Goddard, 2001; Tschannen-Moran et al., 2014). For example, a school atmosphere created by a high level of collective teacher efficacy will produce great normative press for the teachers to persist in committing their educational efforts in the school to achieve desired results (Goddard and Goddard, 2001). The teachers who do not conform to the school norms will experience of social press and have to act congruently with the shared beliefs of the school so as to avoid their actions being sanctioned by group members (Goddard et al., 2000). The continuing influence of the working norms of a school will consequently make teachers internalize their beliefs about the school to be self-referential, which can lead to an increase in the teachers’ identification with the school as well as their willingness to contribute to the school’s objectives (Cybulski et al., 2005).

The empirical finding of the positive impact of collective teacher efficacy on organizational commitment suggests that collective teacher efficacy can be taken as a school orientation and concern for increasing teachers’ motivated commitment to school organization. This provides supports for the idea of developing a mechanism that can improve teachers’ collective efficacy beliefs to develop an effective school. For educational researchers and practitioners who are looking for strategies to promote teachers’ commitment to schools, interventions can target the whole school through executing measures (e.g., offering school support as discussed the current study) that can lead to the improvement of teachers’ collective teacher efficacy beliefs. In addition, this finding contributes to understanding the profiles of schools in which the teachers are relatively “at risk” of leaving schools. That is, more attention needs to be paid to those schools with low levels of collective teacher efficacy beliefs because the teachers in such schools are less likely to have a strong identity and bond with the schools. Overall, this finding extends the influence of collective teacher efficacy to an aspect of teachers’ attitudes toward their schools, which has received little attention thus far, and makes a notable contribution by documenting the positive effect of collective teacher efficacy on teacher commitment.

### 6.3. Pathway from school support to organizational commitment *via* collective teacher efficacy

In this investigation, we sought to investigate collective teacher efficacy as the mechanism through which collective teacher efficacy contributes to organizational commitment. Our results appeared to show the significant mediating effect of collective teacher efficacy on the relationship between school support and organizational commitment. This finding implies that the teachers who are working in a school that is in the presence of sufficient staff support tend to hold strong collective efficacy beliefs and therefore are more likely to identify with the mission and goals of the schools, psychologically attach to the schools, and engage in their work. To some extent, this confirms that the reinforcing the normative school environment by offering sufficient staff support raises in-school distinctiveness and makes the identification and commitment of teachers to the school easier (Dumay and Galand, 2012). This finding is a step forward in uncovering the process through which school contextual factors (e.g., school support in this study) influence teachers’ organizational commitment. Collective teacher efficacy offers alternative explanations for why the schools which provide high levels of staff support also demonstrate high levels of organizational commitment. The significant mediating role of collective teacher efficacy further suggests that collective teacher efficacy deserves more attention of the school leaders (Cansoy et al., 2020; Qadach et al., 2020; Karacabey et al., 2022). Specifically, a leader should be aware of the characteristic of collective efficacy of his or her school and conscious of the measures that can lead to the improvement of teachers’ collective teacher efficacy beliefs. In addition, developing a supportive school environment can be taken as an effective way for schools to overcome low levels of collective teacher efficacy beliefs, which will lead to the enhancement of their teachers’ commitment to schools.

### 6.4. Implications for practice: Fostering a supportive school culture

The findings of our studies, which shed light on the influences of school support on collective teacher efficacy and organizational commitment, carry significant practical implications concerning building schools for school leaders “who are tasked with creating contexts conducive to teacher and student work” (Tschannen-Moran et al., 2014, p. 303). The results of the current study highlighted the importance of a focus upon building a supportive school environment to facilitate teachers’ instructional practices so as to improve their collective efficacy beliefs about their school’s capability of improving students’ achievement, which is predictive of the differences in teachers’ organizational commitment among schools. From a practical point of view, school leaders are encouraged to invest more effort in firmly building an enabling school by fostering a supportive school culture to empower and motivate teachers to extend themselves and give their best at school.

A supportive school should be a professional learning community, which is characterized as collectives focusing on productive collaboration among teachers, de-privatized teaching practices, and reflection (Yin et al., 2019), for teachers to bolster their collective efficacy beliefs and commitment (Lee et al., 2011; Tschannen-Moran et al., 2014). A supportive school environment should supply the staff with not only hardware but also the opportunities for professional development through thoughtfully planned professional development and data-based decision making (Ware and Kitsantas, 2007). A school acting as a professional learning community provides a normative climate where teachers can seek help from school leaders and colleagues, acquire teaching strategies, and jointly solve problems and conduct instructional innovation (Adams and Forsyth, 2006; Liu et al., 2022). The teachers in a school characterized by a professional learning community will therefore tend to develop high levels of beliefs in their school’s capability to substantiate student learning grounded in these joint experiences (Lee et al., 2011; Moolenaar et al., 2012; Liu et al., 2022). Such collegial environment, in which teachers experience greater work interdependence and less isolation, will result in a greater sense of collective enterprise shared by the teachers and hence enhance their willingness to exert more effort toward achieving the common school goals.

The advice that was documented by an emergent body of research (e.g., Goddard et al., 2004; Tschannen-Moran and Hoy, 2007; Goddard and Salloum, 2012) suggested that, to develop a supportive environment in a school, the critical role that is played by the school leaders can be emphasized. As supportive leaders, school principals should unite the school faculty for their common vision, set the tone for the quality of interactions among teachers, set up the structures to support interactive collaboration among teachers, and articulate and guard norms to foster a strong sense of professionalism (Tschannen-Moran et al., 2014). They should be responsive to teachers’ concerns, encourage and provide firm support to teachers wishing to try new teaching ideas, attempt to address student needs, increase teachers’ control of teaching environment, and offer opportunities to influence school policies (Tschannen-Moran and Hoy, 2007; Ware and Kitsantas, 2007).

### 6.5. Limitations and future research directions

A number of limitations of the present investigation should be noted and can be used to suggest directions for future research. The first limitation is the relatively small school sample, which could constrain the analytical techniques that could be used and the power of the study. Despite a lack of well-established best practices when sample sizes at different levels of analysis are modest, according to Marsh et al. (2012), doubly latent multilevel analysis requires a large effective sample size at the group level (e.g., the school level in this investigation). Future studies should consider replicating the hypotheses with a larger school sample. Alternatively, the Bayesian estimation method, which has been found to be potent in producing accurate results for multilevel CFA with a very small group-level sample size (e.g., 20 groups) in a simulation study (Hox et al., 2012), can be considered. Second, although a prior was decided about the directions of the relationships among the three constructs in the hypotheses, the cross-sectional design cannot essentially serve a validation of the casual nature of these relationships and is unlikely to shed much light on the complex interplay among the three key constructs (Moolenaar et al., 2012). Therefore, the interpretation of the current results which were derived from the correlational data should be offered tentatively and interpreted with cautions (Klassen, 2010). Longitudinal data collection could be reasonably called for in future studies to clarify the causal ordering or directionality of the relationships among the three key variables. Third, although the measurement of teachers’ organizational commitment was drawn from Park (2005), it only contains three items which are incline to assess the organizational cohesion perceived by teachers. Future studies may consider adopting other scales to measure the rich connotations of teachers’ organizational commitment. Last, the absence of the control of other school-level demographic factors (e.g., school SES, school size, school type, school composition, and school achievement history) is another limitation. By controlling important school background variables, the way how school support predicts collective teacher efficacy through its contribution to each of the four remote sources of collective teacher efficacy (Goddard and Goddard, 2001) will be an interesting topic of future studies for researchers who are interested in the mechanism of the influence of school support on collective teacher efficacy.

## 7. Conclusion

The purpose of this investigation is to utilize the doubly latent multilevel method to investigate the relationships among school support, collective teacher efficacy, and organizational commitment. The results identified the mediation mechanism that was played by collective teacher efficacy in explaining the effect of school support on organizational commitment. The finding contributes to offering suggestions for school leaders and policy makers on creating a supportive environment in their schools as an influential approach to strengthen teachers’ collective efficacy beliefs which will be beneficial to promoting their commitment to schools. In other words, if it is wished to improve teachers’ dedication to schools, the current study suggested that efforts emphasizing on creating norms of collective efficacy should become one of the top priorities for school administrators through fostering a supportive school culture. Overall, this investigation provides effective practical suggestions for educational communities who are putting effort in searching for ways to improve teachers’ commitment to schools.

## Data availability statement

The raw data supporting the conclusions of this article will be made available by the authors, without undue reservation.

## Ethics statement

The studies involving human participants were reviewed and approved by Survey and Behavioral Research Ethics Committee, The Chinese University of Hong Kong. The patients/participants provided their written informed consent to participate in this study.

## Author contributions

ZZ, JL, and HY participated in the design of the study and the questionnaire. ZZ performed data analysis and wrote the first draft of the manuscript. HY and XY performed paper drafting and revision. ZZ and XY contributed to the conceptualization of the manuscript and reviewed the content of manuscript. All authors contributed to the article and approved the submitted version.

## Funding

This work was funded by the Project Impact Enhancement Fund (PIEF) 2021–22 of the Faculty of Education, The Chinese University of Hong Kong.

## Conflict of interest

The authors declare that the research was conducted in the absence of any commercial or financial relationships that could be construed as a potential conflict of interest.

## Publisher’s note

All claims expressed in this article are solely those of the authors and do not necessarily represent those of their affiliated organizations, or those of the publisher, the editors and the reviewers. Any product that may be evaluated in this article, or claim that may be made by its manufacturer, is not guaranteed or endorsed by the publisher.
